# Viral haemorrhagic fevers and malaria co-infections among febrile patients seeking health care in Tanzania

**DOI:** 10.1186/s40249-022-00959-z

**Published:** 2022-04-25

**Authors:** Sima Rugarabamu, Susan F. Rumisha, Gaspary O. Mwanyika, Calvin Sindato, Hee-Young Lim, Gerald Misinzo, Leonard E. G. Mboera

**Affiliations:** 1 SACIDS Foundation for One Health, Sokoine University of Agriculture, Morogoro, Tanzania; 2 Department of Veterinary Microbiology, Parasitology and Biotechnology, Sokoine University of Agriculture, Morogoro, Tanzania; 3 Muhimbili University of Health and Allied Sciences, Dar es Salaam, Tanzania; 4 National Institute for Medical Research, Dar es Salaam, Tanzania; 5 Malaria Atlas Project, Geospatial Health and Development, Telethon Kids Institute, Perth, WA Australia; 6 Mbeya University of Science and Technology, Mbeya, Tanzania; 7 National Institute for Medical Research, Tabora Research Centre, Tabora, Tanzania; 8 Korea Disease Control and Prevention Agency, National Institute of Health, Osong, Chungchungbukdo Republic of Korea

**Keywords:** Malaria, Viral haemorrhagic fevers, Febrile illnesses, Co-infection, Tanzania

## Abstract

**Background:**

In recent years there have been reports of viral haemorrhagic fever (VHF) epidemics in sub-Saharan Africa where malaria is endemic. VHF and malaria have overlapping clinical presentations making differential diagnosis a challenge. The objective of this study was to determine the prevalence of selected zoonotic VHFs and malaria co-infections among febrile patients seeking health care in Tanzania.

**Methods:**

This facility-based cross-sectional study was carried out between June and November 2018 in Buhigwe, Kalambo, Kyela, Kilindi, Kinondoni, Kondoa, Mvomero, and Ukerewe districts in Tanzania. The study involved febrile patients seeking health care from primary healthcare facilities. Blood samples were collected and tested for infections due to malaria, Crimean-Congo haemorrhagic fever (CCHF), Ebola virus disease (EVD), Marburg virus disease (MVD), Rift Valley fever (RVF) and yellow fever (YF). Malaria infections were tested using rapid diagnostics tests while exposure to VHFs was determined by screening for immunoglobulin M antibodies using commercial enzyme-linked immunosorbent assays. The Chi-square test was used to compare the proportions.

**Results:**

A total of 308 participants (mean age = 35 ± 19 years) were involved in the study. Of these, 54 (17.5%) had malaria infection and 15 (4.8%) were positive for IgM antibodies against VHFs (RVF = 8; CCHF = 2; EBV = 3; MBV = 1; YF = 1). Six (1.9%) individuals had both VHF (RVF = 2; CCHF = 1; EVD = 2; MVD = 1) and malaria infections. The highest co-infection prevalence (0.6%) was observed among individuals aged 46‒60 years (*P* < 0.05). District was significantly associated with co-infection (*P* < 0.05) with the highest prevalence recorded in Buhigwe (1.2%) followed by Kinondoni (0.9%) districts. Headache (100%) and muscle, bone, back and joint pains (83.3%) were the most significant complaints among those infected with both VHFs and malaria (*P* = 0.001).

**Conclusions:**

Co-infections of VHF and malaria are prevalent in Tanzania and affect more the older than the younger population. Since the overlapping symptoms in co-infected individuals may challenge accurate diagnosis, adequate laboratory diagnosis should be emphasized in the management of febrile illnesses.

## Background

Viral haemorrhagic fevers (VHF) have become more prevalent in sub-Saharan Africa during recent years, causing either sporadic or large-scale epidemics, thus posing significant risk to public health. The VHFs reported in sub-Saharan Africa in recent years include Crimean-Congo haemorrhagic fever (CCHF), Ebola virus disease (EVD), Lassa fever (LF), Marburg virus disease (MVD), Rift Valley fever (RVF), and yellow fever (YF) [1–4]. On the other hand, malaria is among the most important mosquito-borne parasitic endemic disease in the region, accounting for 94% of the reported cases globally [5]. VHFs and malaria are among the major causes of febrile illnesses in humans in sub-Saharan Africa. The fact that malaria prevalence in sub-Saharan Africa is high, it is likely that the prevalence of co-infections of VHFs and malaria to be common in the region.

Of the VHFs, RVF and CCHF outbreaks have been reported in Tanzania [6–8]. Although no clinical case of EVD, MVD or YF have been reported in Tanzania, its geographical position puts the country at high risk. This is due to the fact that outbreaks of the three diseases have been reported from its neighbouring countries [9–12]. Moreover, a recent study in Tanzania has reported the presence of immunoglobulin M (IgM) and IgG antibodies against to CCHF, EVD, MVD, RVF, and YF in 12.8% of the population across the country. The specific disease estimated seroprevalence of CCHF, EVD, MVD, RFV and YF was 2.0%, 3.4%, 1.2%, 4.8%, and 1.4%, respectively [4]. Tanzania is among the 10 countries with the highest malaria cases and deaths, accounting for 3% of the global cases and 5% of global deaths [5]. Malaria is endemic in almost all regions of Tanzania, with over 95% of the population at risk, though with significant transmission variations between and within districts [13, 14]. The disease accounts for about a quarter and a third of all outpatient attendances and hospital admissions, respectively, and is the leading cause of hospital mortality [15].

In sub-Saharan Africa, VHF and malaria co-infections have been reported in Gabon [16], Senegal [17], Nigeria [18], and Democratic Republic of the Congo [19]. In a recent systematic review, prevalence of EVD and malaria co-infection was reported to be between 19 and 72% [20]. Co-infections of malaria and VHF have been described to shape and influence the development of inflammatory diseases, with either beneficial or detrimental outcomes [20–22]. A study in Liberia has indicated that malaria parasitaemia was associated with an increase in the probability of surviving EVD infection [23]. In the contrary, in a study in Sierra Leone, the mortality risk was highest among patients with both EVD and malaria than those with EVD alone [24].

Most VHFs and malaria initially present with similar non-specific symptoms and signs of fever, headache, joint pains and weaknesses, posing challenges in differential diagnosis [19, 25]. Clinical diagnosis of VHF is considered when a patient is from an endemic area, tested malaria negative, and an alternative diagnosis cannot be established. When not systematically investigated, VHFs are often misdiagnosed as malaria due to similar clinical presentations; thus delays the identification of VHF outbreaks and the associated potentially high morbidity and mortality [20]. There is dearth of information on VHF and malaria co-infections in Tanzania and possibly underreported because of the limited number of laboratories capable of diagnosing VHF infections. The objective of this study was therefore to determine the prevalence of co-infections of VHFs and malaria among febrile patients seeking health care in Tanzania.

## Methods

### Study areas and design

**Fig. 1:**
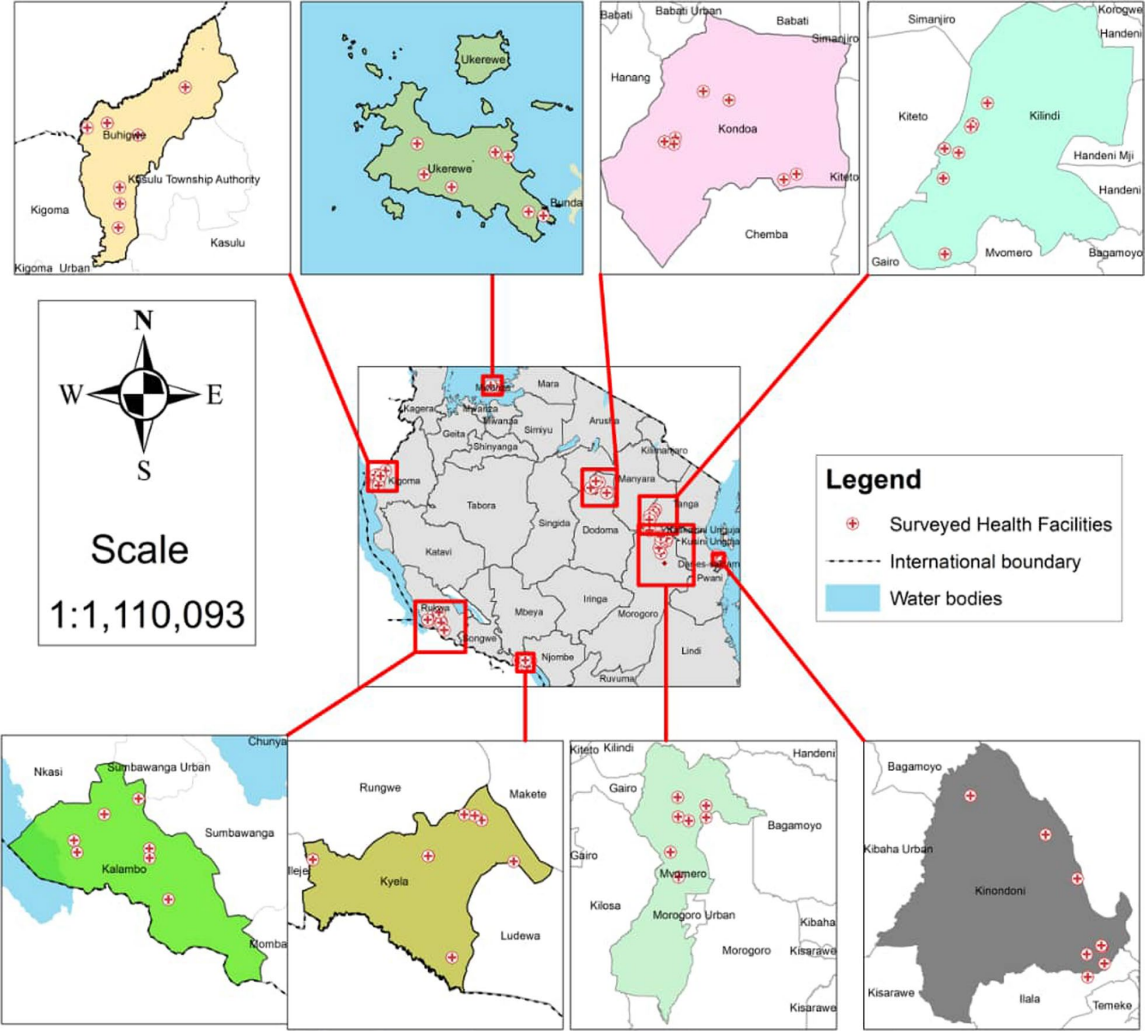
Map of Tanzania showing the ecological zones, study districts and sites

This study was carried out in eight districts in five distinct ecological zones of Tanzania. The zones are Western, Southern-highlands, North-eastern, Central and Lake Victoria. The western zone is characterised by tropical forest, unimodal rainfall pattern, and an altitude of 2300 m. The Southern-highlands zone covers regions with high precipitations, tropical forest, bimodal rainfall pattern, low temperatures and elevation > 2300 m. The North-eastern Tanzania is characterised by a bimodal rainfall pattern and seasons of high temperatures. The Central zone comprises of a semi-arid area at an elevation of about 1000 m characterized by moderate precipitation and unimodal rainfall pattern. The Lake Victoria area is characterized by a bimodal rainfall pattern and intermediate temperatures and an elevation of 1134 m. The study districts were Buhigwe, Kalambo (western), Kyela (southern-highlands), Kilindi, Kinondoni (north-eastern), Kondoa, Mvomero (central) and Ukerewe (Lake Victoria) (Fig. 1). The study area has been described in detail elsewhere [4].

In each district, a hospital and two health centres were conveniently selected while a random sampling was done in selecting four dispensaries. Patients presenting with fever were recruited for the study. Fever was defined as any patient with an axillary temperature of  > 38 ℃ based on clinical thermometer reading on the day of health facility visit. Clinicians from the study team and those from the selected health facilities carried out clinical assessment for each participant. Socio-demographic and epidemiological data were collected from each participant.

### Sample size calculation

To calculate the sample size for this study, the fever prevalence of 25% [13], a desired absolute precision of 5%, and a confidence level of 95% were used [26]. The minimum estimated sample size was 289 individuals. To split among the districts, the population-weighted samples based on the zonal and regional prevalence of the febrile population were used [13]. District specific sample sizes were as follows: Western zone: Buhigwe = 33; Kalambo = 31; Southern-highlands zone (Kyela) = 48; North-eastern zone: (Kilindi = 31; Kinondoni = 39); Central zone: (Kondoa = 32; Mvomero = 49); and Lake Victoria zone: Ukerewe = 45. The sample size considered a contingency of 10% to account for non-response, refusal, or missingness. The district sample was split equally among the study facilities.

### Data collection

This facility-based study was conducted between June and November 2018. The research team was oriented on the study objectives, procedures, research ethics and trained on data collection tools. All febrile patients attending healthcare facility were targeted. A face-to-face interview was conducted to collect information on socio-demographic and clinical characteristics using a semi-structured questionnaire programmed in the *AfyaData* app installed in smartphones [27]. The socio-demographic characteristics collected included age, sex, occupation, village, workplace, and area of residence. Each individual was examined for symptoms and/or clinical features of headache, rash, fatigue, muscle pain, bone pain, back or joint pain, nausea, abdominal pain, bruising, vomiting, red spots (on the skin/eye/mucosa), and jaundice. Each participant was assigned a code that restricted her/his direct identification. Axillary temperature was recorded using a digital clinical thermometer. Five millilitres of blood were collected from adults and children > 10 years and 2 ml from children < 10 years of age by venepuncture using standard sterile technique. Malaria infection was tested using malaria rapid diagnostic tests (CareStart™ malaria HRP-2/pLDH, American Access Bio Company, USA). The sensitivity and specificity of CareStart™ malaria HRP2/pLDH (Pf/pan) combo test are high [28] and test has been approved for diagnosis of malaria at the point of care in Tanzania.

Sera were harvested from the collected blood samples by centrifugation at the laboratories in the respective district hospitals. The samples were labelled using a unique identification number, archived, and stored in liquid nitrogen (− 196 ℃) before being transported to the laboratory at Sokoine University of Agriculture in Morogoro, Tanzania, where they were stored at −80 ℃ until examination. Aliquots of the sera were tested for the presence of human IgM antibodies against CCHF, EVD, LF, MBV, RVF, and YF using commercial enzyme-linked immunosorbent assays (ELISA) kits (My BioSource, Inc., San Diego, CA, USA), according to the manufacturer's instructions. To validate the ELISA, determination of the intra-assay coefficient of variation (CV), inter-assay CV, recovery, linearity and parallelism was performed. The intra-assay CV (%) and inter-assay CV (%) were less than 15% for reactive samples.

### Data analysis

Data were analysed using Statistical Package for the Social Sciences (SPSS) Statistics version 23 (IBM, Armonk, NY, USA). Frequencies, median, and associated interquartile ranges (IQRs) were calculated. The Chi-square test (or Fisher’s exact test where appropriate) was used to compare the proportions of positive malaria, VHF and co-infections frequency by sex and age. A statistically significant difference was considered when the *P*-value was below 0.05. Cut-off values for ELISA antibody analysis were determined following the manufacturer’s instructions. The assay performance was considered adequate when the intra- and inter-assay coefficient of variabilities were less than 15%.

## Results

### Socio-demographic and clinical characteristics

A total of 308 participants were involved in the study. Of these, 185 (60.1%) were females, and 123 (39.9%) males. The mean age was 35 ± 19 years. Over half (54.2%) of participants were in the 16‒45 years age category. About two-thirds (62.3%) of participants had primary school education. Crop farmers accounted for the largest proportion (43.2%) of the participants (Table 1).

**Table 1:** Socio-demographic and clinical characteristics of study participants

Variable	Description	Frequency	Percentage (%)
Sex	Female	185	60.1
	Male	123	39.9
Age (years)	0‒15	49	15.9
	16‒30	86	27.9
	31‒45	81	26.3
	46‒60	55	17.9
	≥ 61	37	12.0
Education	None	77	25.0
	Primary	192	62.3
	Secondary	29	9.4
	College	10	3.3
Occupation	Crop farming	133	43.2
	Employed	44	14.4
	Trading	21	6.8
	Livestock keeping	20	6.4
	Student	27	8.8
	Others	63	20.4
Symptom/sign	Headache	242	48.4
	Muscle, bone, back, or joint pain	160	32.0
	Abdominal pain	82	16.4
	Fatigue	45	9.0
	Rashes	31	6.2
	Vomiting	25	5.0
	Nausea	18	3.6
	Jaundice	7	1.4
	Bleeding/bruising	3	0.6
	Red spots on the skin/eye/mucosa	2	0.4

The most common symptoms among the study participants were body aches and pains which included headache (48.4%), muscle, bone, back, or joint pain (32.0%), and stomach pain (16.4%). The least observed signs were jaundice, bleeding/bruising, and red spots on the skin, eyes or mucosae (Table 1).

### Seroprevalence of malaria or viral haemorrhagic fevers

Of the 308 febrile individuals, 54 (17.5%) were positive to the rapid diagnostic malaria test. The prevalence of malaria infection was higher among females (10.1%; *n* = 31) than males (7.5%; *n* = 23). Individuals aged 0‒15 years (6.8%; *n* = 21) were most affected by malaria. However, there was no significant difference between sex and the age groups (*P* > 0.05) (Table 2). A total of 15 (4.8%) participants were positive for IgM antibodies against VHF tested (RVF = 8; CCHF = 2; EBV = 3; MBV = 1; YF = 1). None of the samples was positive for Lassa fever virus antibody. There was a significant difference between the age groups among seropositive individuals to EVD (*P* < 0.05). Seroprevalence of RVF, CCHF, MVD and YF were similar in all age groups (Table 2).

**Table 2:** Seroprevalence of single infection of malaria, RVF, CCHF, EVD, MVD, and YF by age and sex

Variable	Malaria		RVF		CCHF		EVD		MVD		YF	
	*n* (%)	*P*-value	*n* (%)	*P*-value	*n* (%)	*P*-value	*n* (%)	*P*-value	*n* (%)	*P*-value	*n* (%)	*P*-value
Age (years)												
0‒15	21 (6.8)	0.69	0		0		0		1 (0.3)		0	
16‒30	14 (4.5)		1 (0.3)		1 (0.3)		0		0		0	
31‒45	12 (3.9)		3 (1.0)	0.96	1 (0.3)	0.67	0	0.015	0	0.99	1 (0.3)	0.99
46‒60	7 (2.3)		1 (0.3)		0		3 (0.9)		0		0	
≥ 61	0 (0.0)		3 (1.0)		0		0		0		0	
Sex												
Female	31 (10.1)	0.56	4 (1.3)	0.67	1 (0.3)	0.53	1 (0.3)	0.35	0	0.21	1 (0.3)	0.44
Male	23 (7.4)		4 (1.3)		1 (0.3)		2 (0.6)		1 (0.3)		0	

Key: *RVF* Rift Valley fever, *CCHF*  Crimean-Congo Haemorrhagic fever, *EVD* Ebola Virus Disease, *MVD* Marburg Virus Disease, *YF* Yellow fever

### VHFs and malaria co-infections

Six (1.9%) individuals had both VHF (RVF = 2; CCHF = 1; EVD = 2; MVD = 1) and malaria infections. Of those with VHF and malaria co-infections, four (66.7%) were males and two (33.3%) were females. In relation to age, the highest co-infection rate (0.6%), was observed among individuals aged 46–60 years (*P* < 0.05). Location was significantly associated with co-infection (*P* < 0.05) as higher prevalence was recorded among those from Buhigwe (1.2%) followed by Kinondoni (0.9%) districts (Table 3).

**Table 3:** Seroprevalence of malaria, RVF, CCHF, EVD, MVD, YF and the respective co-infections with malaria (Mal) by district, *n* (%)

District	Malaria	RVF	RVF + Mal	CCHF	CCHF + Mal	EVD	EVD + Mal	MVD	MVD + Mal	YF	YF + Mal
Buhigwe	3 (0.9%)	1 (0.3%)	1 (0.3%)	0	0	2 (0.6%)	2 (0.6%)	1 (0.3%)	1 (0.3%)	0	0
Kinondoni	6 (1.9%)	2 (0.6%)	1 (0.3%)	1 (0.3%)	1 (0.3%)	0	0	0	0	1 (0.3%)	0
Mvomero	4 (1.3%)	1 (0.3%)	0	0	0	0	0	0	0	0	0
Kyela	14 (4.5%)	0	0	0	0	0	0	0	0	0	0
Kalambo	10 (3.2%)	1 (0.3%)	0	0	0	0	0	0	0	0	0
Kondoa	3 (0.9%)	1 (0.3%)	0	0	0	1 (0.3%)	0	0	0	0	0
Kilindi	2 (0.6%)	1 (0.3%)	0	0	0	0	0	0	0	0	0
Ukerewe	12 (3.9%)	1 (0.3%)	0	1 (0.3%)	0	0	0	0	0	0	0

Key: *RVF* Rift Valley fever, *CCHF* Crimean-Congo Haemorrhagic fever, *EVD* Ebola Virus Disease, *MVD* Marburg Virus Disease, *YF* Yellow fever, *Mal* malaria

Severe headache was the most frequent (100%) complain among those with both VHF and malaria infections. Other aches, including muscle, bone, back, and joint pains were reported by 83.3% of those with co-infections of VHFs and malaria (Table 4).

**Table 4:** Main clinical characteristics of malaria, VHF and VHF + malaria co-infections

Symptoms and sign	Number of subjects (%)			*P*-value
	Malaria	VHF	VHF + Malaria	
Headache	8 (14.8%)	1 (7%)	6 (100%)	0.001
Aches and pains	7 (12.9%)	0	5 (83.3%)	0.001
Vomiting	2 (3.7%)	0	2 (33%)	0.32
Rashes	0	1 (7%)	1 (16.6%)	0.31
Fatigue	3 (5.5%)	1 (7%)	1 (16.6%)	0.23
Abdominal pain	2 (3.4%)	0	1 (16.6%)	0.44
Bleeding/bruising	0	0	1 (16.6%)	0.23
Jaundice	0	0	1 (16.6%)	0.24

*VHF* Viral haemorrhagic fever

## Discussion

Malaria was the most important febrile associated infection in the study districts. Overall, about 5% of the study population were positive for IgM antibodies against VHF tested, with RVF accounting for the largest proportion of the infections. Co-infections of malaria and VHFs were detected in about 2% of the febrile individuals seeking care from health facilities. While RVF outbreaks have been reported to occur in low malaria endemic districts of Tanzania [29–32], CCHF has been reported in high malaria area of the country [6, 7]; thus allowing the occurrence of mixed infections in patients as previously reported in other endemic regions [33]. The prevalence of malaria infections in the current study was lower than that reported by other recent studies in Tanzania [13, 14, 33]. This could be due to the differences in the study period, targeted population and also biases of health-facility based surveys. A relatively higher prevalence of co-infection of malaria and EVD has been reported from studies during outbreaks in Democratic Republic of the Congo [19] and Sierra Leone [25]. Similar to the findings of a study in Nigeria [34], no malaria and YF co-infection was observed in this study.

The VHF and malaria co-infection rate observed in this study was associated with older age. The higher prevalence of VHF and malaria co-infections among the adults is likely to be associated with higher exposure compared with children due to their livelihoods activities that bring them closer to sources of VHF infection [35]. Many studies have linked VHF and malaria co-infections with increased human activity around ecological zones of increasing biodiversity, climate change, negative healthcare-seeking practices of some community members, gate-keeping role by traditional healers and traditional health care system, water and sanitation challenges, burial practices, and corpse management capacity in poor-resource settings [22, 25, 36, 37].

VHFs (EVD, MVD, RVF) and malaria co-infections were most prevalent in Buhigwe district in Kigoma Region of western Tanzania, bordering the Democratic Republic of Congo. Available statistics indicate that Buhigwe is among the districts with high malaria prevalence (24%) in Tanzania [14, 38]. Moreover, a recent seroprevalence study has reported a 4.4% overall prevalence of VHF in the same district, the highest rate reported in Tanzania [4]. Co-infections due to malaria and either RVF or CCHF were most prevalent in Kinondoni district. The district is within the high malaria burden region [38] and a recent study has reported an overall prevalence of VHF of 2.8% [4].

Aches and pains including severe headache were significantly noted in most of all the co-infected patients in this study compared to patients with VHF alone. However, this study could not determine whether malaria, VHF or both to be responsible for the observed symptoms. Our study agrees with others that patients with VHFs usually present with non-specific signs, including fever, vomiting, diarrhoea, fatigue, myalgia and headache [19]. Most often, later on, if not self-recovered, the disease progresses to rashes and some affected individuals will show evidence of coagulation disorders with multiple foci of mucosal haemorrhage and persistent bleeding, while massive haemorrhages usually only occur in fatal cases [39]. Our study observed an overlap presentation of fever, headache, muscle/bone/back/joint pain, and vomiting in all patients co-infected with VHF and malaria.

The presence of antibodies against RVF, CCHF, EVD, MVD and malaria in this study was highly unexpected. The results are alarming because if sublethal VHF infections commonly occur, they are likely to be missed in co-infections with malaria.

This study has limitations including use of rapid test to diagnose malaria, which could result to some cases been missed due to low parasitaemia. In addition, no information on the use of antimalarials prior to the test was obtained which could result to false positives. The study was conducted between June and November, a period associated with both high and low malaria season depending on the district, which could influence visits to health facilities and availability of febrile patients as treatment seeking is low during low malaria seasons, and levels of malaria risk obtained. The serological results are based on antibody detection which are likely to be a reflection of cumulative exposure and/or antibody persistence following single or multiple exposure. Moreover, point of care tests also have limitations associated with possibility that one or more as-yet-unknown pathogens are circulating among the human populations sampled in this study, producing antibodies cross-reactive. The results of this study should be interpreted with caution. Despite useful information gathered on the presence of VHF and malaria co-infections, the findings from this study are based on very small sample hence weaken the accuracy and may not be representative.

## Conclusions

The findings of this study show that the prevalence of co-infections of viral haemorrhagic fevers and malaria in Tanzania is generally low, though higher rates were observed among the older individuals. Since infections due to malaria and VHF are associated with similar symptoms, these findings suggest the need for improvement in case management of febrile illnesses based on adequate laboratory diagnosis. It is equally important that more studies are needed to establish how VHFs and malaria infections interact to improve case management and control in the future. The findings also highlight the need to conduct molecular studies to identify specific pathogens.

## Data Availability

The datasets generated for this study are available on request to the corresponding author.
